# RETRACTED ARTICLE: Summer Research Training Provides Effective Tools for Underrepresented Minorities to Obtain Doctoral Level Degrees

**DOI:** 10.1007/s40615-016-0330-0

**Published:** 2017-01-11

**Authors:** Oluwatoyin A. Asojo, Ashish Damania, Teri L. Turner, Gayle Slaughter, Kendal D. Hirschi

**Affiliations:** 1 National School of Tropical Medicine, Baylor College of Medicine, Houston, TX 77030 USA; 2 Center for Research, Innovation and Scholarship in Medical Education, Texas Children’s Hospital, Department of Pediatrics, Baylor College of Medicine, Houston, 77030 TX USA; 3 Graduate School, Baylor College of Medicine, Houston, 77030 TX USA; 4 Children’s Nutritional Research Center, Baylor College of Medicine, Houston, 77030 TX USA

The editor-in-chief has recommended retraction due to irregularities with the reporting of the primary data set. The online version of this article contains the full text of the retracted article as electronic supplementary material.

## Electronic supplementary material

ESM 1 Former article version (PDF 40615_2016_330_OnlinePDF.pdf FILESIZE 995 KB)

