# Supplementary material for: RETRACTED ARTICLE: Summer Research Training Provides Effective Tools for Underrepresented Minorities to Obtain Doctoral Level Degrees
Source: J Racial Ethn Health Disparities. 2017 Jan 11;4(6):1224. doi: 10.1007/s40615-016-0330-0 (PMC5705752; doi:10.1007/s40615-016-0330-0)
Supplement: Supplementary file 1 — Former article version (PDF 40615_2016_330_OnlinePDF.pdf FILESIZE 995 KB) [file 40615_2016_330_MOESM1_ESM.pdf]

J. Racial and Ethnic Health Disparities
DOI 10.1007/s40615-016-0330-0

CrossMark

Summer Research Training Provides Effective Tools for Underrepresented Minorities to Obtain Doctoral Level Degrees

Oluwatoyin A. Asojo[1] · **Ashish Damania**[1] · **Teri L. Turner**[2] · **Gayle Slaughter**[3] · **Kendal D. Hirschi**[4]

RETRACTED ARTICLE

Received: 20 November 2016 / Revised: 17 December 2016 / Accepted: 19 December 2016
© The Author(s) 2017. This article is published with open access at Springerlink.com

Abstract The ethnic, racial, and cultural diversity of the USA is not reflected in its healthcare and biomedical workforce. Undergraduate research programs are used to encourage underrepresented minorities to pursue training for biomedical careers, but there is limited published data on doctoral degree completion rates by participants in these programs. This study evaluates the attainment of doctoral degrees by a NIH-funded cohort of undergraduates from the 1998–2002 Baylor College of Medicine's Summer Medical Research Training (SMART) program. Variables collected included race, ethnicity, gender, tier status of undergraduate institution, and terminal degree status. The majority of cohort participants were underrepresented minorities, while the rest were socio-economically underserved, or first generation college students. Of the 163 participants, 65 (56%) were confirmed to have completed doctoral level biomedical or healthcare degrees: 48 MDs (41%), 22 PhDs (19%), 4 MD/PhDs (3%), and 1 PharmD (1%). There was no statistical significant difference in doctoral degree attainment based on gender, ethnicity, or tier status of participants. The completion rates of doctoral degrees by underrepresented minority participants (65%) in this program exceeds that of underrepresented minority graduate students (38%) and that of non-minority graduate students (51%) nationally based on National Science Foundation data for US citizens and permanent residents, suggesting that this 9-week summer research experience provides a pathway for underrepresented minorities to successfully attain the doctoral degrees required for careers in the health sciences.

Keywords Training to diversify the health-care work-force · SMART program · Summer undergraduate research programs · Recruitment efforts · Evaluation

This study was completed as partial fulfillment of requirements for the Baylor College of Medicine's Master's Teacher Fellowship Program by OAA and KDH.

✉ Oluwatoyin A. Asojo
asojo@[illegible].edu

[1] National School of Tropical Medicine, Baylor College of Medicine, Houston, TX 77030, USA

[2] Center for Research, Innovation and Scholarship in Medical Education, Texas Children's Hospital, Department of Pediatrics, Baylor College of Medicine, Houston 77030, TX, USA

[3] Graduate School, Baylor College of Medicine, Houston 77030, TX, USA

[4] Children's Nutritional Research Center, Baylor College of Medicine, Houston 77030, TX, USA

Introduction

African Americans, Latinos, and indigenous people make up over 30% of the US population but less than 9% of health and biomedical professions, and this low percentage is problematic since a culturally competent and diverse workforce is an integral part of eradicating health disparities [1]. Additionally, the percentage of underrepresented minorities graduating with doctoral degrees in science, technology, engineering, and mathematics (STEM) is likewise low, increasing slightly from ~9.5 to ~10.9% from 2003 to 2008 [2]. Interventions to increase the diversity of doctoral level scientists include undergraduate research experiences [3–13]. These research experiences are either part-time during the school year or full-time during the summer and offer the opportunity to learn new skills and develop an appreciation for applications of scientific concepts that were introduced in the classroom while learning the scientific skills required to think as a scientist [3–5, 14–16].

Table 1 Participant description and demographics

(*n* 116)	All participants (*n* 116)	Choice in research area	No choice in research area
College type			
Tier 1	43 (37%)	31 (27%)	12 (10%)
Non-tier 1	73 (63%)	36 (31%)	37 (32%)
Race/ethnicity			
American Indian	2 (2%)	1 (1%)	1 (1%)
Asian	7 (6%)	7 (6%)	0
African American	47 (41%)	27 (23%)	20 (17%)
Hispanic (Latino/a)	46 (40%)	21 (18%)	25 (22%)
Pacific Islander	4 (3%)	1 (1%)	3 (3%)
Unknown	8 (7%)	8 (7%)	0
White	2 (2%)	2 (2%)	0
Gender			
Female	70 (60%)	39 (34%)	31 (27%)
Male	46 (40%)	28 (24%)	18 (16%)
Total number of participants	116 (100%)	67 (58%)	49 (42%)

Despite the integration of undergraduate research experiences into many STEM undergraduate programs and extensive investments from federal, state, and private organizations into these programs, there is only limited published data on doctoral degree completion by participants of these programs, due to an absence of funding to carry out extensive longitudinal evaluation studies [9, 17, 18]. Some published evaluations use admission to graduate or professional school as yardsticks of success despite data from the National Science Foundation that reveals a persistent and significant attrition rate for attaining doctoral level degrees in STEM fields by underrepresented minorities. Specifically, only 38.1% of enrolled underrepresented minorities attained their PhDs as compared to 51.3% of nonminorities [19, 27]. Others including the highly successful Meyerhoff scholarship program at the University of Maryland Baltimore County, which significantly increased the likelihood of pursuing doctoral degrees in STEM, focus on academic year long STEM Undergraduate Research Programs [20–23].

In an effort to close the missing gap in the literature, a goal-based Tylerian model evaluation of the 1998–2002 cohort of the Summer Medical and Research Training (SMART) Program at the Baylor College of Medicine (BCM) was performed, with the objective to determine doctoral level advanced degree completion by underrepresented minorities as well as to conduct a sub-analysis on the impact of choice or self-efficacy on long-term outcomes. This evaluation was completed after sufficient time had elapsed to allow participants to complete doctoral level degrees.

Methods

Analysis Framework

A goal-based Tylerian model was used as a framework to investigate the long-term doctoral degree completion of the 1998–2002 cohort of the SMART program. This cohort allowed sufficient time for attaining the principal goal of this summer research program, which was training students to successfully achieve doctoral-level degrees in biomedical sciences. Furthermore, this time framed allowed a sub-analysis on choice and doctoral degree completion.

Setting and Participants

Participant description and demographics are listed in Table 1. Baylor College of Medicine (BCM) is one of the top ranked medical schools in the USA and is located within the Texas Medical Center, the world's largest medical metropolis. BCM has four schools: the medical school, graduate school, school of allied health, and the national school of tropical medicine, which all offer professional and post-baccalaureate degrees and diplomas. BCM does not offer any undergraduate degrees. With over a century of biomedical research, BCM has been a top 20-funded NIH-funded medical school since 2006 with $205,461,582 in NIH funding in 2015.

The participants received funding from the National Institute of General Medical Sciences (NIGMS R25 GM56929) or the National Heart, Lung, and Blood Institute (NHLBI HL07480). Sub-analysis on choice as a

Table 2

Specific objectives of SMART program
• To promote independent functioning as scientists
• To expand and strengthen each participant's base of scientific knowledge
• To help students improve preparation for the GRE
• To provide personal career counseling
Activities involved in SMART program
• SMART participants are placed in research laboratories for 9 weeks where they become a part of the research team, learn new techniques, and contribute to the research effort
• Mentors receive training about the goals of the SMART program and becoming effective mentors.
• Students develop projects and present results at the end of the summer
• Students participate in research discussion and seminar series. ~25% of speakers are underrepresented faculty members
• Students have access to the SMART GRE preparatory course
• Students have social activities and movie nights that introduce them to peers and other mentors, while creating a supportive environment
• Students live as a community in the Rice University dormitories
• Students had opportunities to shadow physicians and interact with diverse groups of scientists both formally and informally

RETRACTED ARTICLE

RETRACTED ARTICLE

Table 3 Degrees obtained by SMART participants

Degree obtained	NIGMS/NHLBI	Latino/African American/other	Gender (M/F)	Tier 1/non-tier 1	Total
MD	24/24	20/19/9	20/28	20/28	48
PhD	13/9	9/8/5	12/10	5/17	22
MD/PhD	4/0	2/2/0	1/3	3/1	4
PharmD	0/1	0/0/1	1/0	0/1	1
MS	5/0	2/2/1	3/2	3/2	5
MPH	1/0	0/1/0	1/0	0/1	1
MBA	0/1	1/0/0	0/1	0/1	1
MPA MA	1/0	0/1/0	0/1	0/1	1
No advanced degree	2/4	3/3/0	2/4	2/4	6
Status unknown	17/10	9/11/7	6/21	10/17	27
Total	67/49	46/47/23	46/70	43/73	116

measure of self-efficacy was facilitated because participants funded by the NIGMS could choose a research program of interest (from >22 research programs at Baylor College of Medicine), while those funded by the NHLBI were limited to specific research mentors involved in heart, lung, or blood research. To minimize the effects of other variables such as career development activities, the participants were chosen from overlapping years because these students were involved in identical activities through the SMART program.

Program Description

The SMART program was developed to provide frontier-level, biomedical summer research projects for undergraduates in a supportive environment with supplemental educational activities (Table 2).

Variables

Independent variables investigated included type of undergraduate school (tier 1 or not), gender, race, and ethnicity of participant. Dependent variables examined were the completion of doctoral and other post-graduate degree. The variable investigated for a sub-analysis was choice in research assignment.

Data Processing

Selected accumulated data corresponding to the participants funded by NIGMS and NHLBI from 1998 to 2002 was extracted from the SMART database (see supplementary methods). Advanced Degree was defined as Masters and above. Doctoral degrees were defined to include PhDs or equivalent degrees, medical degrees or equivalent degrees, or combinations of both. Additional data processing is described in supplementary methods. Tier 1 versus non-tier 1 colleges was identified with the Carnegie Classification of Institutions of Higher Education's listing of very high research activity (RU/VH) universities (see supplementary methods).

IRB

Institutional Review Board (IRB) approval was obtained for this study from Baylor College of Medicine (H-34910).

Table 4 Choice in research and degree completion

Degree type	No choice in research (NHLBI) (%)	Choice in research (NIGMS) (%)
MD	49	36
PhD	18	19
MD/PhD	0	6
PharmD	2	0
Total biomedical doctoral degrees	69	61
Total advanced degree	71	71
No advanced degree	8	3
Status unknown	20	25

Table 5 Tier status and degree completion

Degree type by Tier	Tier 1 (%)	Non-tier 1 (%)
MD	47	38
PhD	12	23
MD/PhD	7	1
PharmD	0	1
Total biomedical doctoral degrees	66	63
Total advanced degree	73	70
No advanced degree	4	5
Status unknown	23	23

RETRACTED ARTICLE

Table 6 Gender and degree completion

Degree type obtained by gender	Male (%)	Female (%)
MD	43	40
PhD	26	14
MD PhD	2	4
PharmD	2	0
Total biomedical doctoral degrees	73	59
Total advanced degree	82	65
No advanced degree	4	6
Status unknown	13	30

Results

Terminal degrees completed by participants are listed in Table 3 while Tables 4, 5, 6, and 7 list the percentages of students that attained degrees based on the independent variables.

Discussion

Success Regardless of Choice of Research Area

A sub-analysis was performed to determine if the ability to choose the discipline of their research projects impacted the attainment of doctoral degrees by participant. The challenges to completing doctoral degrees are well known and studied [24]; however, on a fundamental level, it is believed that aspects of the goal (doctoral degree) that make achievement of the goal important to the individual and the individual's self-efficacy (for example, choice) play a role in attaining goals [25]. The confirmed advanced degree outcomes for participants from both programs are summarized in Table 3. Similar percentages of students in both cohorts completed doctoral level biomedical degrees (Table 4), whereas a greater percentage of those who had no choice did not complete any advanced degree (Fig. 1). A statistical analysis of completion of doctoral level or advanced degrees by participants with and without choice in research revealed no statistical differences between the two groups (Tables 8 and 9).

Table 7 Race/Ethnicity and degree completion

Degree type obtained	African American (%)	Hispanic (Latino/a) (%)	Other*
MD	40	43	39%
PhD	17	19	22%
MD PhD	4	4	0
PharmD	0	0	4%
Total biomedical doctoral degrees	61	66	65%
Total advanced degree	70	73	70%
No advanced degree	6	7	0%
Status unknown	23	20	30%

Undergraduate Institution Type and Doctoral Degree Completion

Access to undergraduate research opportunities varies across different types of tertiary institutions. Tier 1 and research-intensive universities typically have the ability to provide students the exposure and opportunities to interact with faculty conducting state-of-the-art research. Undergraduate students at tier 1-type research-intensive universities expect to have access to research opportunities, and these colleges are ranked by US News and World reports based on this access. Conversely, such opportunities are not as readily available at schools that are not research intensive [26]. Since the students participating in the summer research programs were from diverse types of undergraduate institutions, the data was probed to determine if attending a tier 1 vs. a non-tier 1 undergraduate school correlated with increased advanced degree completion rates. A larger percentage of participants from both cohorts attended non-tier 1 schools, and for some of these students, this summer program was their first exposure to research outside their classes. Despite this difference in educational background, similar percentages of participants from tier 1 and non-tier 1 schools successfully completed doctoral level degrees (Fig. 1, Table 5). There are no statistically significant differences in doctoral or advanced level degree completion between participants from tier 1 and other schools (Tables 8 and 9).

Gender and Doctoral Degree Completion

Gender imbalances in STEM fields are well known, and overall, more American men complete doctorate and masters level degrees than women. Interestingly, the situation differs for underrepresented minority women who attain doctorate degrees at higher rates than underrepresented minority men [27, 28]. Male and female participants had high biomedical sciences doctoral rates (Table 6), and there was no statistical difference in post-graduate degree completion by gender (Fig. 1, Tables 8 and 9). The gender analysis reveals one of the major limitations in long-term follow-up studies—the difficulty tracking subjects, especially after more than a decade since participation in a program, and females were harder to track than men.

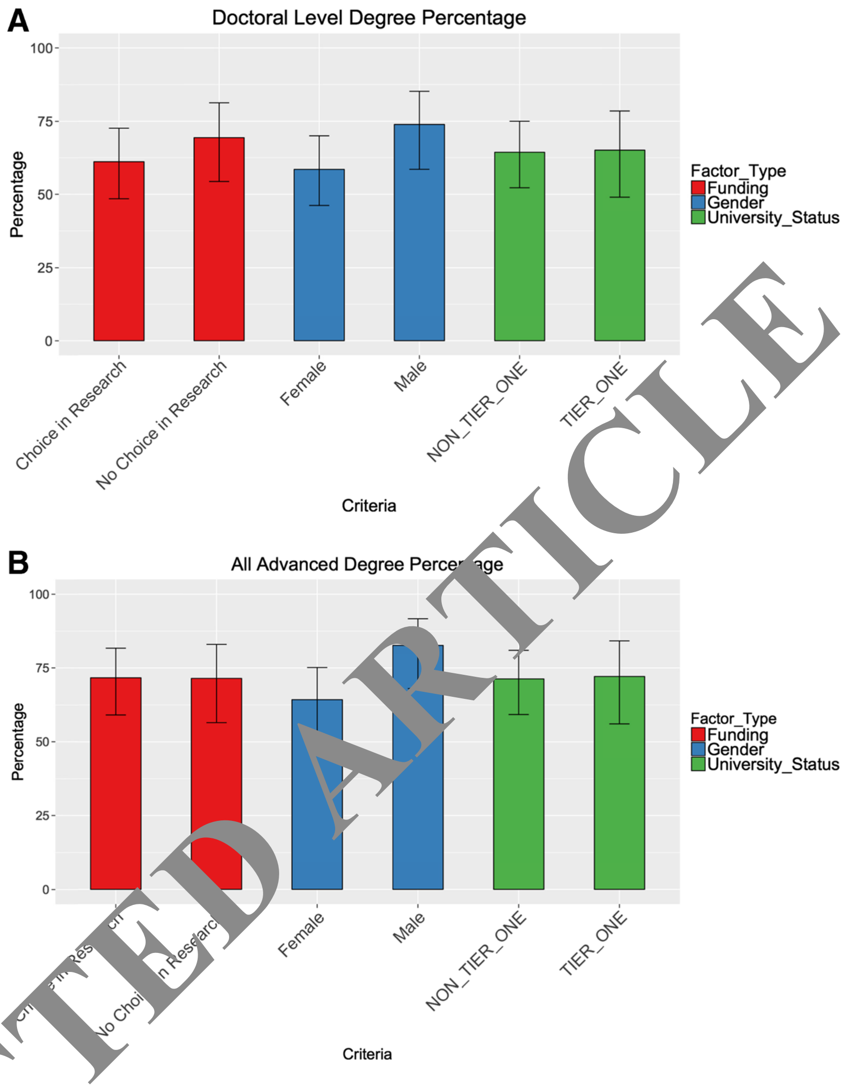

Fig. 1 **a** Doctoral level degree completion percentage. **b** All advanced degree completion percentage

RETRACTED ARTICLE

Race, Ethnicity, and Doctoral Degree Completion

A major objective of this summer program is to increase the numbers of underrepresented minorities with doctoral level degrees in the biomedical sciences. This cohort of participants reveals high numbers and percentages of doctoral degrees by participants that self-identified as African American or Latino/a (Tables 3 and 7). Another limitation to the more detailed analysis of the ethnicity and race was the small numbers of white students in the cohort, since the program targeted mostly underrepresented minorities. Instead, members of underrepresented groups were compared to participants that were not underrepresented minorities, and there was no statistical difference in completion of advanced degrees (Fig. 1, Tables 8

Table 8 Statistical analysis of doctoral level degree completion

Doctoral degree completion by	Chi-square (*P* value)	Fisher's test (*P* value)	Kruskal-Wallis rank sum test (*P* value)	Cohen's effect size	Cliff's delta
Latino/a) versus not Latino/a	0.80	1	0.91	−0.02 (negligible)	−0.01 (negligible)
African American versus not African American	0.54	0.55	0.43	−0.17 (negligible)	−0.06 (negligible)
Male versus female	1	1	0.86	−0.03 (negligible)	−0.01 (negligible)
Choice in research area versus no choice	0.57	0.57	0.51	0.14 (negligible)	0.05 (negligible)
Tier 1 versus non-tier 1	1	1	0.91	−0.02 (negligible)	−0.01 (negligible)

Participants without confirmed degree were excluded

Table 9 Statistical analysis of advanced level advanced degree

Advanced degree completion by	Chi-square (*P* value)	Fisher's test (*P* value)	Kruskal-Wallis rank sum test (*P* value)	Cohen's effect size	Cliff's delta
Latino/a versus not Latino/a	0.67	0.69	0.67	−0.09 (negligible)	−0.02 (negligible)
African American versus not African American	0.69	0.68	0.62	−0.10 (negligible)	−0.03 (negligible)
Male versus female	0.68	0.67	0.56	−0.12 (negligible)	−0.03 (negligible)
choice in research area versus no choice	0.42	0.40	0.24	−0.25 (small)	−0.06 (negligible)
Tier 1 versus non-tier 1	1	1	0.84	−0.04 (negligible)	−0.01 (negligible)

Participants without confirmed degree were excluded

RETRACTED ARTICLE

and 9). Furthermore, underrepresented minority SMART participants do not have the high attrition rate observed in the general population for attaining doctoral level degrees in STEM fields; instead, they complete PhDs and other advanced at comparable rates as other students.

Comparison with the Meyerhoff Program

As mentioned previously, one of the limitations of this analysis is the large numbers of lost participants. This analysis was conservative and treated lost participants as non-degreed. If less conservative analysis were performed and missing participants were discarded, 84% of the SMART participants completed doctoral degrees while 94% completed some advanced Masters level degree. Regardless of the methodology, the outcomes support previous observations that undergraduate research experiences enhance the likelihood of attaining advanced biomedical degrees [6, 8, 11, 13]. Furthermore, doctoral degree completion percentages for the SMART program are comparable to the enrollment percentages of the most successful year-long research programs on record for the Meyerhoff scholarship program at the University of Maryland Baltimore county [20–22]. The Meyerhoff program is considered the national model of a successful program and 68.6% of the 1996–2005 cohort entered either a doctoral level degree in the STEM or MD [20]. The Meyerhoff Scholars program takes a holistic approach to ensuring that scholars achieve a research-based PhD degree and includes comprehensive training program that offers summer research, tutoring, mentorship, and other support over the 4 years of undergraduate studies [20–22]. This work goes further by tracking the completion of advanced degrees and not just admission into graduate programs. The SMART program is similar to the Meyerhoff program that it has mentorship support and creates a community environment over a single summer. The SMART community is integrated within the BCM community of over 100 underrepresented minority post-baccalaureate, PhD, MD/PhD, and post-doctoral scholars.

Conclusion

A majority of members of this cohort of the SMART program (65%) completed doctoral level degrees in the biomedical sciences. This analysis was undertaken at least 12 years after participating in the SMART program, allowing sufficient time to complete doctoral level degrees. A conservative assessment was undertaken and only counted as degreed are those participants for whom conclusive evidence was obtained regarding degree completion. The analysis presented here is just a small portion of the extensive data available from the more than two decades of undergraduate research training at the Baylor College of Medicine. Funding is required to probe the entire SMART database to identify specific factors and variables that lead to the success of this 9-week summer research program.

Supplementary Methods

The SMART database is stored using Filemaker Pro database software, and it contains information on all participants in SMART since its inception. Relevant data was exported into a text file after removing all personal identifying information. The data text file was edited to ensure consistent textual definitions for variables and exported into SQLite database tables for further analysis (https://www.sqlite.org).

Tier 1 versus non-tier 1 colleges were identified with the Carnegie Classification of Institutions of Higher Education's listing of very high research activity (RU/VH) universities http://carnegieclassifications.iu.edu/downloads/cc2010_classification_data_file.xls. The list of RU/VH universities was downloaded as a CSV file, and the classification was added into the SQLite database table. SQL queries were made for final analysis. Chi-Square test was used to determine the significance of the differences between variables. Data from the SQLite was queried through RSQLite R package to get the data into R data frame for statistical analysis. Participants that we could not verify their completion of advanced degrees were treated as status unknown.

Link to NSF data http://www.nsf.gov/statistics/2015/nsf15311/digest/theme4.cfm#minorities.

Acknowledgements The authors would like to thank Ms. Kerri Mejia, Mr. Nathaniel Wolf, Dr. Geeta Singhal, and the staff of the Master Teacher Fellowship Program.

Compliance with Ethical Standards

Funding No funding was received for preparing the manuscript. The cohorts studied were from two NIH-funded programs, and participants received funding from the National Institute of General Medical Sciences (NIGMS R25 GM56929) or the National Heart, Lung, and Blood Institute (NHLBI HL07480) and Gayle Slaughter was PI of both grants. Kendal Hirschi was funded in part by National Science Foundation award 1557890.

Conflict of Interest The authors declare that they have no conflict of interest.

Ethical Approval Institutional Review Board (IRB) approval was obtained for this study from Baylor College of Medicine (H-34910). All procedures performed in studies involving human participants were in accordance with the ethical standards of the institutional and/or national research committee and with the 1964 Helsinki declaration and its later amendments or comparable ethical standards.

Open Access This article is distributed under the terms of the Creative Commons Attribution 4.0 International License (http://creativecommons.org/licenses/by/4.0/), which permits unrestricted use, distribution, and reproduction in any medium, provided you give appropriate credit to the original author(s) and the source, provide a link to the Creative Commons license, and indicate if changes were made.

References

1. Andrulis DP, Siddiqui NJ, Purtle JP, Duchon L. [illegible] Protection and Affordable Care Act of 2010: Advancing Health [illegible] for Racially and Ethnically Diverse Popula[illegible]ons, Joint Center for Political and Economic Studies Washingt[illegible] DC 20005, 2010.
2. Sowell R, Allum J, Okahana H, Doctora[illegible]itiative on minority attrition and completion., Washin[illegible]ton, DC: [illegible]cil of Graduate Schools, Council of Graduate Scho[illegible]hington DC, 2015;82.
3. Lopatto D. Survey of undergraduate [illegible]earc[illegible] experiences (SURE): first findings. Cell Biol [illegible]004;3:[illegible]70–7.
4. Weaver GC, Russell [illegible] W[illegible] Inquiry-based and research-based laboratory [illegible]pedagog[illegible] in undergraduate science. Nat Chem Biol. 2008;4:57[illegible]80.
5. Eagan Jr MK, Sha[illegible]ess J, Hurtado S, Mosqueda CM, Chang MJ. Engagin[illegible]dergradu[illegible] in science research: not just about faculty willin[illegible]ness. Res High Educ. 2011;52:151–77.
6. Thiry [illegible]eston [illegible]J, Laursen SL, Hunter AB. The benefits of multi-[illegible] resea[illegible]xperiences: differences in novice and experienced stu[illegible]ents' reported gains from undergraduate research. CBE Life Sci [illegible]12;11:260–72.
7. [illegible]uske AJ, Wilson J, Walls M, Clarke B. Experiences of mentors training underrepresented undergraduates in the research laboratory. CBE Life Sci Educ. 2013;12:403–9.
8. Hanson MJ. Introducing ethics to chemistry students in a "research experiences for undergraduates" (REU) program. Biochem Mol Biol Educ. 2015;43:76–80.
9. Cameron C, Collie CL, Chang S. Introducing students to cancer prevention careers through programmed summer research experiences. J Cancer Educ. 2012;27:233–42.
10. Burnette JM, Wessler SR. Transposing from the laboratory to the classroom to generate authentic research experiences for undergraduates. Genetics. 2013;193:367–75.
11. Adedokun OA, Parker LC, Childress A, Burgess W, Adams R, Agnew CR, Leary J, Knapp D, Shields C, Lelievre S, Teegarden D. Effect of time on perceived gains from an undergraduate research program. CBE Life Sci Educ. 2014;13:139–48.
12. Teegarden D, Lee JY, Adedokun O, Childress A, Parker LC, Burgess W, Nagel J, Knapp DW, Lelievre S, Agnew CR, Shields C, Leary J, Adams R, Jensen JD. Cancer prevention inter[illegible]isciplinary education program at Purdue University: overview [illegible] and preliminary results. J Cancer Educ. 2011;26:626–32.
13. Hernandez PR, Schultz PW, Estrada M, Woodcock A, C[illegible]e P[illegible]C. Sustaining optimal motivation: a longitudina[illegible] analysis of in[illegible]erventions to broaden participation of underrep[illegible]ented s[illegible]dents in STEM. J Educ Psychol. 2013;105(1):[illegible]32. doi:[illegible]037/a0029691.
14. Shaffer CD, Alvarez C, Baile[illegible] C, Barnard D, Bhalla S, Chandrasekaran C, Chandrasekara[illegible]V, Chun[illegible] HM, Dorer DR, Du C, Eckdahl TT, Poet JL, [illegible]hlich[illegible] Goodman AL, Gosser Y, Hauser C, Hoopes LL, Jo[illegible]son [illegible]Jones CJ, Kaehler M, Kokan N, Kopp OR, Kuleck GA, McNeil G, [illegible]ss R, Myka JL, Nagengast A, Morris R, Ove[illegible]voo[illegible] PJ, Shoop E, Parrish S, Reed K, Regisford EG, Revie D, R[illegible]wald AG, Saville K, Schroeder S, Shaw M, Sku[illegible]e [illegible]mith C, Smith M, Spana EP, Spratt M, Stamm J, Thomps[illegible]JS, [illegible]ersik M, Wilson BA, Youngblom J, Leung W, Buhler J, [illegible]rdis ER, Lopatto D, Elgin SC. The genomics educ[illegible]ation partne[illegible]: successful integration of research into laboratory [illegible] at a diverse group of undergraduate institutions. CBE Life Sc[illegible]duc. 2010;9:55–69.
15. Characteristics of Excellence in Undergraduate Research (COEUR) The council on undergraduate research, 2012.
16. [illegible]ussell SH, Hancock MP, McCullough J. The pipeline. Benefits of [illegible]ndergraduate research experiences. Science. 2007;316:548–9.
1[illegible]. Senn B, Kirsch M, Sanz CC, Karlou C, Tulus K, De Leeuw J, Ringner A, Goossens GA, Cleary V. How cancer research could benefit from the complex intervention framework: students' experiences of the European academy of nursing science summer school. Eur J Cancer Care (Engl). 2011;20:1–4.
18. Whitt KJ. Experiences from the National Institute of Nursing Research: Summer Genetics Institute 2004. Policy Polit Nurs Pract. 2005;6:15–6.
19. Allen-Ramdial SA, Campbell AG. Reimagining the pipeline: advancing STEM diversity. Persistence, and Success, Bioscience. 2014;64:612–8.
20. Maton KI, Pollard SA, McDougall Weise TV, Hrabowski FA. Meyerhoff scholars program: a strengths-based, institution-wide approach to increasing diversity in science, technology, engineering, and mathematics. Mt Sinai J Med. 2012;79:610–23.
21. Stolle-McAllister K, Sto Domingo MR, Carrillo A. The Meyerhoff way: how the Meyerhoff scholarship program helps black students succeed in the sciences. J Sci Educ Technol. 2011;20:5–16.
22. Carter FD, Mandell M, Maton KI. The Influence of on-campus, academic year undergraduate research on STEM PhD outcomes: evidence from the Meyerhoff scholarship program. J Educ Eval Policy Anal. 2009;31(4):441–462.
23. Maton KI, Sto Domingo MR, Stolle-McAllister KE, Zimmerman JL, Hrabowski III FA. Enhancing the number of African Americans who pursue STEM PhDs: Meyerhoff scholarship program outcomes, processes, and individual predictors. J Women Minor Sci Eng. 2009;15(1):15–37. doi:10.1615/JWomenMinorScienEng.v15.i1.20.
24. Pyhältö K, Toom A, Stubb J, Lonka K. Challenges of becoming a scholar: a study of doctoral Students' problems and well-being. ISRN Education. 2012;2012:12. doi:10.5402/2012/934941.

RETRACTED ARTICLE

25. Locke EA, Latham GP. Building a practically useful theory of goal setting and task motivation. A 35-year odyssey. Am Psychol. 2002;57(9):705–17. doi:10.1037/0003-066X.57.9.705.
26. Snow AA, DeCosmo J, Shokai SM. Low-cost strategies for promoting undergraduate research at research universities, peer review Spring. 2010;12(2). https://www.aacu.org/publications-research/periodicals/low-cost-strategies-promotingundergraduate-research-research.
27. Women, minorities, and persons with disabilities in science and engineering: 2103, National Science Foundation, Arlington, VA, 2013. https://www.nsf.gov/statistics/wmpd/2013/.
28. Broadening participation in STEM Graduate Education, American Institutes for Research, Washington, DC, 2014.

RETRACTED ARTICLE
